# Intelligent prediction of RBC demand in trauma patients using decision tree methods

**DOI:** 10.1186/s40779-021-00326-3

**Published:** 2021-05-24

**Authors:** Yan-Nan Feng, Zhen-Hua Xu, Jun-Ting Liu, Xiao-Lin Sun, De-Qing Wang, Yang Yu

**Affiliations:** 1 Department of Transfusion Medicine, The First Medical Center of Chinese PLA General Hospital, No. 28, Fuxing Rd., Beijing, 100853 China; 2 Beijing Hexing Chuanglian Health Technology Co., Ltd., Beijing, 100176 China

**Keywords:** Mathematical model, Intelligent prediction, Decision tree, Non-invasive parameters, Invasive parameters, Trauma, Transfusion

## Abstract

**Background:**

The vital signs of trauma patients are complex and changeable, and the prediction of blood transfusion demand mainly depends on doctors’ experience and trauma scoring system; therefore, it cannot be accurately predicted. In this study, a machine learning decision tree algorithm [classification and regression tree (CRT) and eXtreme gradient boosting (XGBoost)] was proposed for the demand prediction of traumatic blood transfusion to provide technical support for doctors.

**Methods:**

A total of 1371 trauma patients who were diverted to the Emergency Department of the First Medical Center of Chinese PLA General Hospital from January 2014 to January 2018 were collected from an emergency trauma database. The vital signs, laboratory examination parameters and blood transfusion volume were used as variables, and the non-invasive parameters and all (non-invasive + invasive) parameters were used to construct an intelligent prediction model for red blood cell (RBC) demand by logistic regression (LR), CRT and XGBoost. The prediction accuracy of the model was compared with the area under the curve (AUC).

**Results:**

For non-invasive parameters, the LR method was the best, with an AUC of 0.72 [95% confidence interval (CI) 0.657–0.775], which was higher than the CRT (AUC 0.69, 95% CI 0.633–0.751) and the XGBoost (AUC 0.71, 95% CI 0.654–0.756, *P* < 0.05). The trauma location and shock index are important prediction parameters. For all the prediction parameters, XGBoost was the best, with an AUC of 0.94 (95% CI 0.893–0.981), which was higher than the LR (AUC 0.80, 95% CI 0.744–0.850) and the CRT (AUC 0.82, 95% CI 0.779–0.853, *P* < 0.05). Haematocrit (Hct) is an important prediction parameter.

**Conclusions:**

The classification performance of the intelligent prediction model of red blood cell transfusion in trauma patients constructed by the decision tree algorithm is not inferior to that of the traditional LR method. It can be used as a technical support to assist doctors to make rapid and accurate blood transfusion decisions in emergency rescue environment, so as to improve the success rate of patient treatment.

**Supplementary Information:**

The online version contains supplementary material available at 10.1186/s40779-021-00326-3.

## Background

Trauma accounts for approximately 9% of global deaths [1], and deaths mainly occur within the first 12 h after trauma [2]. The first step in trauma treatment is to control the bleeding as soon as possible, identify the mechanism of trauma, and directly transfer the patients to a nearby trauma treatment institution [3]. Post-traumatic blood loss is a potential and preventable leading cause of death [4]. The core principle of treatment is to identify the risk of haemorrhagic shock as early as possible; meanwhile, fluid resuscitation and blood transfusion are needed to maintain the stability of basic vital signs and haemodynamics [5]. The study found that blood transfusion products pre-hospital within 15 min or 15 min after injury were associated with 24-h mortality (5.6% vs. 20.2%) and 30-day mortality (11.8% vs. 22.9%) compared with delayed or non-transfusion [6]. Delayed blood transfusion can lead to pulmonary complications and death [7]. Several studies have found that RBC transfusion in trauma patients is associated with increased morbidity and mortality [8, 9]. Kotwal et al. [10] found that the death rate of the massive blood transfusion group was significantly lower than that of the non-massive blood transfusion group, especially in severe and extremely severe trauma [injury severity score (ISS) > 15]. However, regardless of the trauma severity, the mortality decreased gradually in the massive blood transfusion group, non-massive blood transfusion group and non-transfusion group, and there was a significant difference. With the increase in blood transfusion, the mortality rate gradually increased during hospitalization [10]. Therefore, blood products should be given early in the pre-hospital transfer to improve the patients’ survival rate after trauma, and then other interventions should occur as soon as possible to strictly control the amount of blood transfused.

At present, there are many studies on traumatic massive blood transfusion, including various trauma scoring systems for on the battlefield and for civilians [11–13], which are used to predict when to initiate massive blood transfusion programmes. However, in recent years, with the improvement of early pre-hospital and hospitalization trauma management measures, the proportion of patients with massive blood transfusion has gradually decreased [14]. For traumatic patients who do not meet the massive blood transfusion standard, there are few studies on the need for blood transfusion. The fifth edition of the European Trauma Guide recommends that the target haemoglobin (Hb) should be maintained at (70–90) g/L [5], which can be used as a reference for blood transfusion needs, but the guideline also suggests that the normal initial test results of Hb may mask bleeding, and it is recommended to use the results of repeated Hb tests as laboratory indicators of bleeding. Therefore, only the results of Hb determine whether to perform a blood transfusion, and the reference value is limited. How to judge the best demand for blood transfusion according to the changing vital signs of trauma patients is a difficult problem for emergency doctors. At present, most of the blood transfusion decisions made by doctors are based on their personal experiences, but there is no feasible and recognized reference standard for different individuals. Transfusion too early will not only waste blood components but also affect the prognosis of patients with excessive blood transfusion [10, 15]. A delayed blood transfusion will lead to haemorrhagic shock, aggravate complications such as hypothermia, acidosis and coagulation dysfunction, and seriously affect the survival rate of patients [16].

We believe that compared with traditional statistical methods, the application of machine learning methods can help us to identify whether patients need a blood transfusion and reduce unnecessary complications caused by delayed transfusion, insufficient blood transfusion or excessive transfusion. Therefore, this study proposes a new method to establish an artificial intelligence mathematical model by retrospective analysis of patients’ vital signs, laboratory tests and other data to assist doctors in quickly making decisions on whether a blood transfusion is needed after trauma and to improve the success rate of patient treatment.

## Methods

### Clinical data

The Emergency Trauma Database of the First Medical Center of Chinese PLA General Hospital is a comprehensive, unidentified dataset containing medical information on 22,491 critically ill patients from January 2014 to January 2018 [17, 18]. All patients were admitted to the Emergency Department. The medical information of 1371 trauma patients who were triaged to a critical rescue room was extracted. The data related to blood transfusion were provided by the clinical blood transfusion intelligent management and evaluation system database established by the Department of Transfusion Medicine of the First Medical Center of Chinese PLA General Hospital [19]. The patients’ information in the two databases associated were uniquely identified with the outpatient number. In the process of data extraction, the original data were completely consistent with the database data through quality control. The Medical Ethics Committee of the Chinese PLA General Hospital waived the requirement for written informed consent.

### Contains variables

Basic information (age, sex, height, weight), diagnosis, admission time, discharge time, after-department track, blood transfusion time, blood transfusion components, RBC infusion volume were collected.

Non-invasive detection parameters include vital signs [heart rate (HR), respiration (R), shock index (SI), systolic blood pressure (SBP), diastolic blood pressure (DBP), blood oxygen saturation (SpO_2_), temperature (T)] and test time, trauma location were collected.

Invasive detection parameters include routine blood test parameters [Hb, haematocrit (Hct), platelet count (PLT), C-reactive protein (CRP), interleukin (IL)-6] and test time; coagulation indicators [prothrombin time (PT), activated partial thromboplastin time (APTT), international standardized ratio (INR), prothrombin activity (PTA), fibrinogen (Fib)] and test time; blood gas test parameters [potential of hydrogen (pH), partial pressure of oxygen (PO_2_), partial pressure of carbon dioxide (PCO_2_), total carbon dioxide (TCO_2_), lactate (Lac), actual bicarbonate (AB), standard bicarbonate (SB), potassium (K)] and detection time; trauma severity classification (first level, second level and third level); endotracheal intubation; and vasoactive drugs were collected.

Construct new variables: For the trauma diagnostic classification, we divided the variables into the fields of trauma type (open trauma, blunt injury) and trauma location (head and neck, upper extremity, lower extremity, chest and abdomen, spine, trunk and pelvis). Examples of 10 patients with their features show in more detail in Additional file 1: Table 1.

Variable dimensionality reduction: To reduce the time and complexity of the model operation, only one variable with a high correlation coefficient was retained, such as Hb and Hct, and only the variable Hct was retained.

Inclusion criteria: (1) patients’ diagnoses were matched or fuzzy matched with “injury”, and (2) patients were triaged from the emergency department to a critical rescue room. Exclusion criteria: (1) patients with non-external trauma, and (2) age < 18 years old.

### Acquisition of variables

The process of obtaining variables included extracting and aggregating variables, cleaning variables, and processing variables.

#### Variable extraction

The numerical variables were extracted directly, including vital sign parameters, laboratory test results, and information related to blood transfusion in the database. The results of the first examination when entering the emergency department were used as variables to predict the demand for blood transfusion. If multiple tests were performed before or after blood transfusion, the results closest to the time blood transfusion were included in the analysis. We used natural language processing to extract effective information from unstructured text variables in the database in advance, such as diagnosis and medical orders. We extracted the variable information from the emergency trauma database and then used the patient’s unique identification as the centre, associated it with the blood transfusion information of the clinical blood transfusion database system, and aggregated it into a record.

#### Variable cleaning

We needed to clean duplicate data and formulate retention principles, such as testing the changes of vital signs many times after entering the emergency department and taking the results of the first test as the key variable to judge whether a blood transfusion occurred, checking the invalid value and establishing the criteria, such as height and weight with − 1, 0, etc., checking the logical relationship among the data, such as the time of admission, the time of laboratory examination, and the blood transfusion start time.

#### Variable processing

(1) Classify variable processing: Convert the variables into a numerical vector and then use it to build models, such as gender and other variables; (2) Unstructured text variable processing: Use the automatic counting word segmentation algorithm in natural language processing to transform words into numerical variables; (3) Construct new variables: Divide the diagnostic information of patients, such as diagnostic details and variable processing of trauma location, into phrases and fields and then count and score the different categories in the target variables and train the model using the learned rules to construct new variables.

### Establishing the model

SPSS 22.0 software (IBM, USA) was used to establish the LR model and CRT model. CRT is supervised analysis technology, which uses the binary classification method to divide the data into two pieces at a time and enter it into the left and right two trees. The root node of the tree is a dependent variable, and the child node is based on the classification variable (parent node). The minimum sample size on the CRT parent node established by the non-invasive parameter is 20, and the child node is 10. The minimum sample size of the CRT parent node for invasive parameters is 50, and the child node is 20. If the sample size on the node does not meet this requirement, the node is a terminal node and will no longer be segmented [20].

XGBoost is a gradient lifting decision tree algorithm provided by the Python language. XGBoost is a supervised learning method and is an integrated learning model that is used for classification analysis (processing discrete data) and regression tree analysis (processing continuous data). The XGBoost algorithm is composed of a loss function and a regular function. The loss function calculates the error between the prediction and the real result, and the loss function is constrained based on the minimum error in the actual calculation. The regular function is used to detect the complexity of the model to avoid overfitting. The loss function and the objective function are given according to the actual situation.

### Statistical analysis

The counting data are described by frequency and percentage [*n* (%)], and the measurement data are expressed by mean and standard deviation [mean (SD)] or median and quartile spacing [median (range)]. The measurement data of the two groups were compared by analysis of variance or Kruskal-Wallis non-parametric test, and the counting data of the two groups were compared by the chi-square test. If *P* < 0.05, the difference was statistically significant.

The LR method was used to screen the significant variables with *P* < 0.05 as independent variables and whether a blood transfusion was used as the dependent variable to establish the model. After the regression coefficient was standardized, the risk factor (*OR*) and 95% CI were used to express the relationship between variables and the occurrence of blood transfusion.

CRT and XGBoost models used the original variables, combined variables or constructed new variables of historical datasets for model training. The historical dataset was randomly divided into an 80% training set and a 20% test set. The model was trained on the training set, and the effect of the model was evaluated on the test set.

**Fig. 1:**
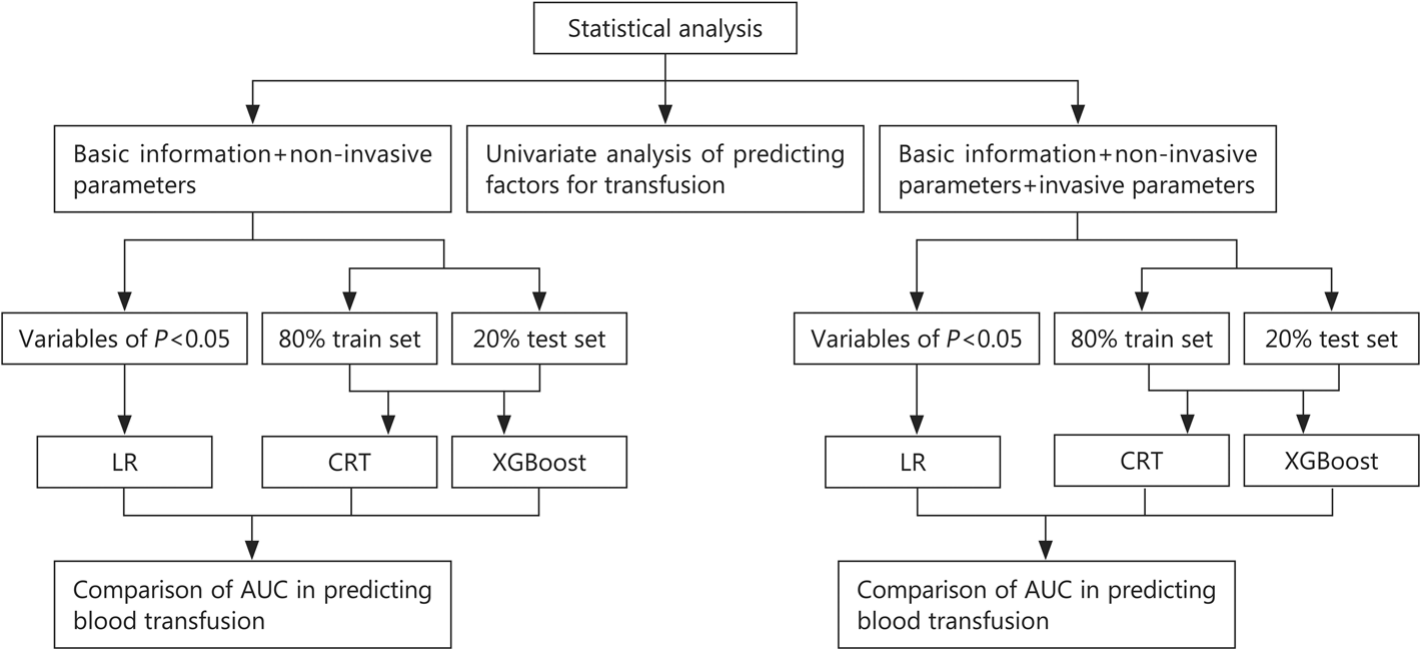
Flowchart of statistical analysis. LR logistic regression, CRT classification and regression tree, XGBoost eXtreme gradient boosting, AUC area under curve

LR, CRT and XGBoost models were compared with whether a blood transfusion was used as the target variable, method 1 (basic information + non-invasive parameters) as analysis variables, and method 2 (basic information + non-invasive parameters + invasive parameters) as analysis variables to establish models, and AUCs were drawn and analyzed. The AUC results of the two methods and three models were compared by *t* test method provided by SciPy library in Python software; if *P* < 0.05, the difference was statistically significant (Fig. 1).

According to the node level (root node, child node) of each variable in the decision tree, the CRT model reflects the importance of each variable. The XGBoost model is represented by the weight of the factors in the tree model of the gradient lifting decision tree algorithm.

## Results

### Patient characteristics

The emergency trauma database of the First Medical Center of Chinese PLA General Hospital contains the medical information of 22,491 critically ill patients. We included 1371 patients who met the study criteria for analysis. Among them, there were 324 females (23.6%) and 1047 males (76.4%). A total of 1183 patients (86.3%) did not receive blood transfusion, and 188 patients (13.7%) received blood transfusion. There were significant differences between the transfusion group and the non-transfusion group in age, HR, SBP, DBP, SI, Hb, Hct, PLT, PT, APTT, PTA, Fib, pH, PO_2_, TCO_2_, Lac, AB, SB, K, endotracheal intubation, vasoactive drugs, trauma location, RBC volume, 24-h RBC and emergency department time (*P* < 0.05). There were no significant differences between the transfusion group and the non-transfusion group in sex, height, weight, R, SpO_2_, T, CRP, IL-6, INR, PCO_2_, trauma severity classification and trauma type (*P* > 0.05) (Table 1).

**Table 1:** Univariate analysis of predicting factors for transfusion

Variable	No-transfusion (*n* = 1183)	Transfusion (*n* = 188)	*P*-value
Age [year, median (range)]	44.00 (29.00, 56.50)	42.00 (28.00, 54.25)	0.049
Gender [*n* (%)]			0.468
Female	284 (24.0)	40 (21.3)	
Male	899 (76.0)	148 (78.7)	
Height [cm, median (range)]^*^	170.00 (164.00, 175.00)	170.00 (163.50, 173.25)	0.242
Weight [kg, median (range)]^*^	68.00 (60.00, 75.00)	67.75 (60.00, 76.00)	0.541
Non-invasive parameters			
HR [beat/min, mean (SD)]^*^	96.95 (24.31)	103.55 (25.87)	0.000
R [beat/min, median (range)]^*^	21.00 (19.00, 23.00)	21.00 (19.00, 26.00)	0.071
SBP [mmHg, mean (SD)]^*^	124.32 (25.28)	117.65 (27.46)	0.000
DBP [mmHg, mean (SD)]^*^	77.58 (15.96)	74.62 (17.83)	0.000
SpO_2_ [%, median (range)]^*^	98.00 (96.00, 99.00)	98.00 (96.00, 99.00)	0.113
SI [mean (SD)]^*^	0.82 (0.29)	0.95 (0.43)	0.000
T [°C, median (range)]^*^	37.00 (36.80, 37.30)	37.00 (36.70, 37.30)	0.389
Invasive detection parameters			
Hb [g/L, median (range)]^*^	126.00 (107.00, 143.00)	107.00 (82.00, 135.00)	0.000
Hct [L/L, median (range)]^*^	3.80 (0.50, 22.00)	0.46 (0.25, 4.00)	0.000
PLT [× 10^9^/L, mean (SD)]^*^	216.81 (94.70)	201.76 (99.53)	0.000
CRP [mg/L, median (range)]^*^	0.95 (0.10, 4.87)	0.41 (0.10, 3.41)	0.806
IL-6 [pg/ml, mean (SD)]^*^	182.37 (380.08)	219.26 (388.41)	0.412
PT [s, median (range)]^*^	14.70 (14.00, 16.00)	15.40 (14.20, 17.08)	0.000
APTT [s, mean (SD)]^*^	37.03 (10.39)	38.50 (12.86)	0.000
INR [median (range)]^*^	15.40 (14.60, 16.40)	15.60 (14.60, 16.60)	0.698
PTA [%, median (range)]^*^	80.00 (68.00, 89.00)	73.50 (61.25, 85.00)	0.000
Fib [g/L, mean (SD)]^*^	3.15 (1.76)	2.72 (1.51)	0.000
pH [median (range)]^*^	1.16 (1.08, 1.28)	1.22 (1.11, 1.39)	0.000
PO_2_ [mmHg, mean (SD)]^*^	120.73 (62.16)	134.12 (73.88)	0.001
PCO_2_ [mmHg, median (range)]^*^	37.00 (33.00, 41.00)	37.00 (32.00, 41.00)	0.116
TCO_2_ [mmol/L, mean (SD)]^*^	24.01 (4.38)	22.66 (4.85)	0.000
Lac [mmol/L, median (range)]^*^	7.41 (7.37, 7.45)	7.39 (7.35, 7.43)	0.000
AB [mmol/L, mean (SD)]^*^	22.88 (4.24)	21.54 (4.73)	0.000
SB [mmol/L, mean (SD)]^*^	23.69 (3.56)	22.39 (4.31)	0.000
K [mmol/L, median (range)]^*^	3.88 (3.56, 4.10)	3.90 (3.60, 4.24)	0.008
Endotracheal intubation [*n* (%)]			0.011
No	1003 (84.8)	145 (77.1)	
Yes	180 (15.2)	43 (22.9)	
Vasoactive drugs [*n* (%)]			0.000
No	1113 (94.1)	151 (80.3)	
Yes	70 (5.9)	37 (19.7)	
Trauma location [*n* (%)]			0.000
Upper extremity	21 (1.8)	3 (1.6)	
Lower extremity	42 (3.6)	7 (3.7)	
Head and neck	386 (32.6)	28 (14.9)	
Chest and abdomen	538 (45.5)	101 (53.7)	
Spine	62 (5.2)	13 (6.9)	
Trunk	20 (1.7)	7 (3.7)	
Pelvis	57 (4.8)	22 (11.7)	
NA	57 (4.8)	7 (3.7)	
Trauma severity classification [*n* (%)]			0.529
First level	999 (84.4)	163 (86.7)	
Second level	179 (15.1)	25 (13.3)	
Third level	5 (0.4)	0	
Trauma type [*n* (%)]			0.921
Open trauma	747 (63.1)	120 (63.8)	
Blunt injury	436 (36.9)	68 (36.2)	
RBC volume [U, median (range)]	0.00	2.00 [0.00, 4.00]	0.000
24 h RBC [U, median (range)]	0.00	2.00 [0.00, 4.00]	0.000
Emergency department time [h, mean (SD)]	23.58 (33.69)	25.61 (37.80)	0.001

^*^Insufficient data and missing. *HR* heart rate, *R* respiration, *SBP* systolic blood pressure, *DBP* diastolic blood pressure, *SpO*_*2*_ blood oxygen saturation, *SI* shock index, *T* temperature, *Hb* haemoglobin, *Hct* haematocrit, *PLT* platelet count, *CRP* C-reactive protein, *IL-6* interleukin-6, *PT* prothrombin time, *APTT* activated partial thromboplastin time, *INR* international standardized ratio, *PTA* prothrombin activity, *Fib* fibrinogen, *pH* potential of hydrogen, *PO*_*2*_ partial pressure of oxygen, *PCO*_*2*_ partial pressure of carbon dioxide, *TCO*_*2*_ total carbon dioxide, *SPO*_*2*_ oxygen saturation, *Lac* lactate, *AB* actual bicarbonate, *SB* standard bicarbonate, *K* potassium, *RBC* volume of red blood cell transfusion, *24 h RBC* the volume of 24 h red blood cell transfusion, *N* number, *NA* not available

### Model prediction

#### Method 1

**Fig. 2:**
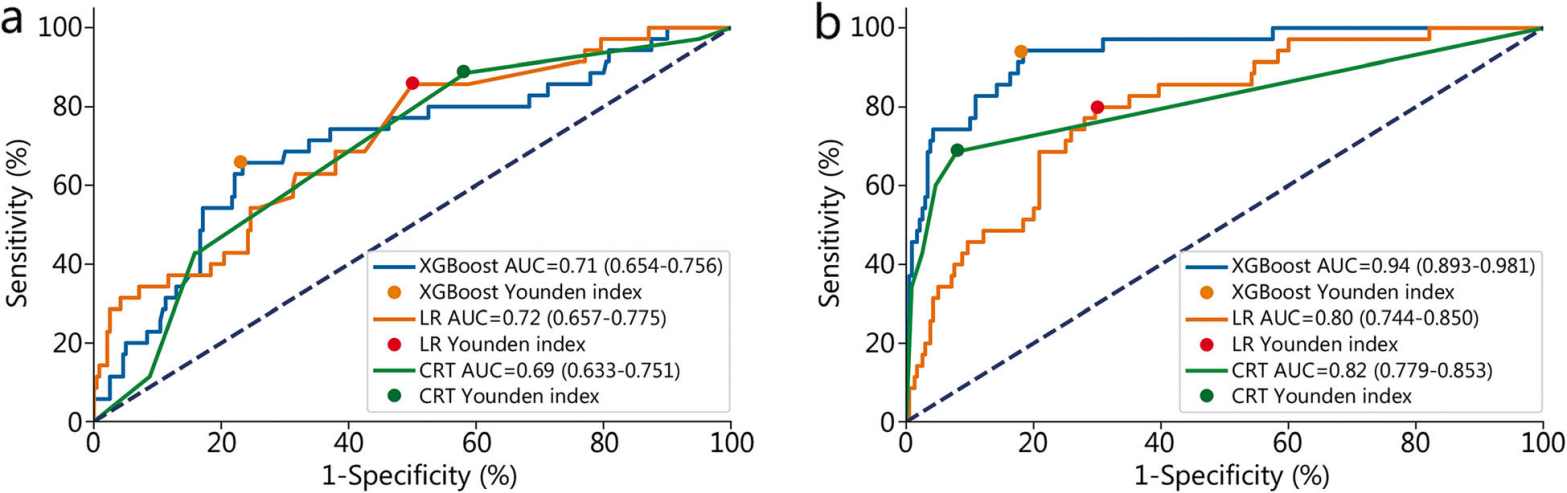
Comparison of AUC between LR, CRT and XGBoost models in predicting blood transfusion. **a** Non-invasive parameters to predict. **b** All parameters to predict. AUC area under curve, XGBoost eXtreme gradient boosting, LR logistic regression, CRT classification and regression tree

The model established with non-invasive parameters predicted the need for blood transfusion after trauma. The AUC of LR model was 0.72 (95% CI 0.657–0.775), which was higher than that of the XGBoost model (0.71, 95% CI 0.654–0.756) and the CRT model (0.69, 95% CI 0.633–0.751) (Fig. 2a). There was a significant difference in the AUC among the three models (*P* < 0.05). The accuracy of the XGBoost model was 0.75, which was higher than that of LR model (0.55) and CRT model (0.48).

#### Method 2

The model established with all parameters was used to predict the need for blood transfusion after trauma. The AUC of the XGBoost model was 0.94 (95% CI 0.893–0.981), which was higher than that of the CRT model (0.82, 95% CI 0.779–0.853) and the LR model (0.80, 95% CI 0.744–0.850) (Fig. 2b). There was a significant difference in the AUC among the three models (*P* < 0.05). The accuracy of the CRT model is 0.89, which is higher than that of XGBoost model (0.83) and LR model (0.72) (Table 2).

**Table 2:** Comparison between the LR, CRT and XGBoost models in predicting blood transfusion

Parameter type	Methods	AUC	Sensitivity	Specificity	Accuracy	Youden index	*P*-value
Non-invasive parameters	XGBoost	0.705	0.66	0.77	0.75	0.19	< 0.001
	LR	0.716	0.86	0.50	0.55	0.12	
	CRT^*^	0.692	0.89	0.42	0.48	0.16	
All parameters	XGBoost	0.937	0.94	0.82	0.83	0.10	< 0.001
	LR^#^	0.797	0.80	0.70	0.72	0.12	
	CRT^#&^	0.816	0.69	0.92	0.89	0.09	

^*^Non-invasive parameter prediction, there was a significant difference in the AUC between CRT and the XGBoost model (*P* < 0.05)

^#^All parameter prediction, there was a significant difference in the AUC between LR and the XGBoost model (*P* < 0.05), and there was a significant difference in the AUC between CRT and the XGBoost model (*P* < 0.05)

^&^All parameter prediction, there was a significant difference in the AUC between CRT and the LR model (*P* < 0.05)

AUC area under the curve, XGBoost eXtreme gradient boosting, LR logistic regression, CRT classification and regression tree

### Variable importance analysis

#### Predicting blood transfusion with non-invasive detection parameters

**Fig. 3:**
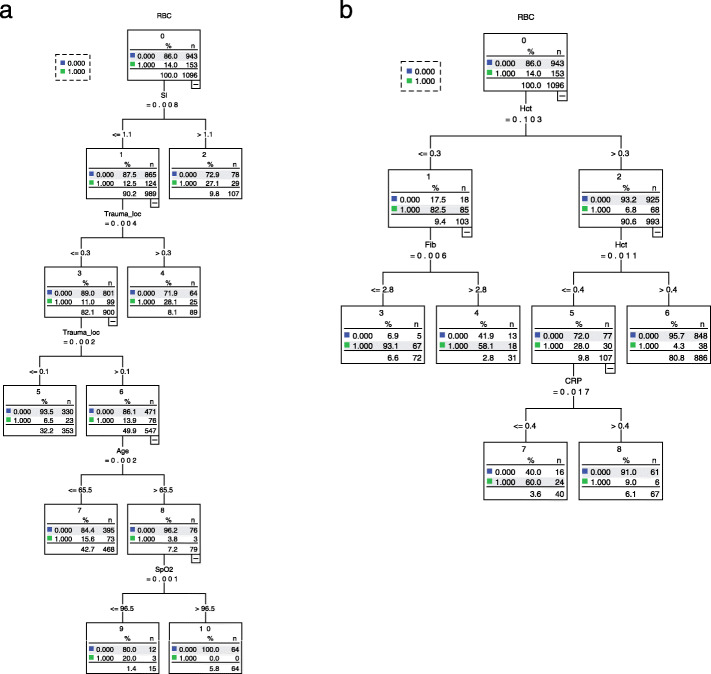
CRT model analysis for predicting transfusion. **a** Non-invasive parameters for prediction. **b** All parameters for prediction. RBC red blood cell, SI shock index, Trauma_loc trauma location, SpO_2_ blood oxygen saturation, Hct hematocrit, Fib fibrinogen, CRP C-reactive protein, CRT classification and regression tree

**Fig. 4:**
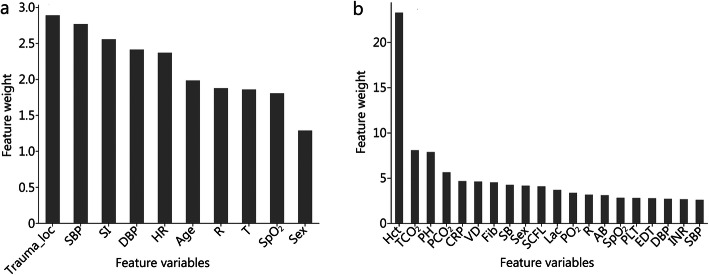
Feature importance ranked by weight in the transfusion prediction model. **a** Non-invasive parameters for prediction. **b** All parameters for prediction. Trauma_loc trauma location, SBP systolic blood pressure, SI shock index, DBP diastolic blood pressure, HR, heart rate, R respiration, T temperature, SpO_2_ blood oxygen saturation, Hct hematocrit, TCO_2_ total carbon dioxide pH potential of hydrogen, PCO_2_ partial pressure of carbon dioxide, CRP C-reactive protein, VD vasoactive drugs, Fib fibrinogen, SB standard bicarbonate, SCFL severity classification in first level, Lac lactate, PO_2_ partial pressure of oxygen, AB actual bicarbonate, PLT platelet count, EDT emergency department time, INR international standardized ratio

LR analysis showed that trauma location (*OR =* 18.371, 95% CI 4.019–83.931, *P* < 0.05) and SI (*OR* = 3.463, 95% CI 1.763–6.801, *P* < 0.05) were risk factors for predicting blood transfusion (Additional file 1: Table 2). The results of the CRT model analysis show that the order of importance of the variables was SI, trauma location, age and SpO_2_ (Fig. 3a). The top five variables in the XGBoost model were trauma location, SBP, SI, DBP and HR (Fig. 4a).

#### Predicting blood transfusion with all test parameters

LR analysis showed that trauma location (*OR* = 7.961, 95% CI 1.422–44.567), vasoactive drugs (*OR* = 2.039, 95% CI 1.092–3.808), PLT (*OR* = 0.995, 95% CI 0.992–0.998), PTA (*OR* = 0.975, 95% CI 0.964–0.988), Hct (*OR* = 0.923, 95% CI 0.899–0.948), SB (*OR* = 0.898, 95% CI 0.844–0.957) and Fib (*OR* = 0.789, 95% CI 0.674–0.924) were risk factors for blood transfusion (*P* < 0.05) (Table 3). The results of the CRT model analysis showed that the order of importance of the variables was Hct, Fib and CRP (Fig. 3b). The top five variables in the XGBoost model were Hct, TCO_2_, pH, PCO_2_ and CRP (Fig. 4b).

**Table 3:** Binary logistic regression analysis for predicting transfusion with all (non-invasive + invasive) parameters

Variable	*OR*	95% CI	*P*-value
SB	0.898	0.844–0.957	0.001
Hct	0.923	0.899–0.948	0.000
VD	2.039	1.092–3.808	0.025
Trauma location	7.961	1.422–44.567	0.018
PTA	0.975	0.964–0.988	0.000
SpO_2_	1.023	0.977–1.071	0.323
PLT	0.995	0.992–0.998	0.001
Fib	0.789	0.674–0.924	0.003

*CI* confidence interval, *SB* standard bicarbonate, *Hct* haematocrit, *VD* vasoactive drugs, *PTA* prothrombin activity, *SpO*_*2*_ blood oxygen saturation, *PLT* platelet count, *Fib* fibrinogen, *OR* odds ratio

## Discussion

In our study, non-invasive detection parameters and all parameters were established to predict blood transfusion in trauma patients, and the decision tree algorithm (CRT and XGBoost) was compared with the traditional statistical method (LR). The results showed that the LR model with basic information and non-invasive parameters was the best, but the sensitivity of the CRT model was the highest, and the specificity and accuracy of the XGBoost model were the highest. The AUC of the basic information + non-invasive parameter + invasive parameter model was higher than that of the non-invasive parameter model. The XGBoost model was the best, and the sensitivity was the highest, but the CRT model had the highest specificity.

AUC embodies the classification ability of the model. LR had the best classification ability in non-invasive parameter prediction, but it was suitable for data analysis and could not be used in clinical applications. The decision tree algorithm had its advantages, and the CRT model had the highest sensitivity and the best ability to identify patients who needed blood transfusion. The specificity and accuracy of the XGBoost model were the highest, and the ability to identify blood transfusion/non-transfusion was the best. When predicting all the parameters, the XGBoost model was the best, and the ability to identify blood transfusion was the best. The CRT model had the best ability to identify transfusion/non-transfusion. The results showed that the more parameters there are, the more prominent the advantages of the decision tree model. The non-invasive parameters can be quickly obtained after trauma patients have obtained medical resources, and the input data can be used to quickly feedback the results of whether the patients need blood transfusion by using the decision tree model. Although the prediction efficiency is slightly lower than all parameters, its time advantage is incomparable. Moreover, trauma is accompanied by changes in blood loss and fluid volume, and vital signs are complex and changeable. The detection time of invasive parameters is approximately 1 h. When the results are obtained, they no longer reflect the current physiological parameters of the patients. Therefore, the non-invasive parameters obtained at any time can reflect the vital signs of patients at that time, and the model can be used to predict at any time, which is convenient for clinical application. When predicting all the parameters, the blood transfusion decisions made by clinicians based on experience are often not accurate. In the case of covering as many data and variables as possible, through a part of the data as a training set, on the basis of learning the experience of clinicians, the machine learning method can more accurately and digitally assist doctors in the decision support of blood transfusion for trauma patients.

Trauma treatment should account for the mechanism of the trauma (open trauma or blunt injury), the location of the trauma (head, chest, etc.), pre-hospital resources, hospital emergency room settings (I, II, etc.) and trauma centre facilities (immediate detection equipment and resources) [21]. Similarly, this study found that when predicting non-invasive parameters, the trauma location and SI had the greatest impact on blood transfusion. The model established by combining age, sex, pre-hospital SI, admission HR, Hb and SpO_2_ can better predict blood transfusion 3 h before admission [22]. The post-traumatic SI is important in assessing the need for blood transfusion and can predict the demand for massive blood transfusion, laparotomy and mortality [23]. The shock index is more sensitive than the ABC score in predicting traumatic massive blood transfusion [24].

Among the predictive variables of all parameters, Hct had a great influence on blood transfusion in the three models. Consistent with our study, many models or scoring systems use Hct as the main parameter for the prediction of traumatic massive blood transfusion [12, 13, 25], which is also consistent with the recommendation that Hb repeat test results should be used as a laboratory indicator of bleeding [5]. Different models have different parameters that affect whether a blood transfusion is carried out. The LR model judges the influence of variables on blood transfusion by risk factors, and the results are generally recognized clinically. Except for trauma location and Hct, vasoactive drugs, PLT, PTA, and Fib were risk factors for blood transfusion demand. The study found that the use of vasoactive drugs can improve vital signs [26], and early routine medication can improve the effective rate of treatment of patients with severe trauma. Traumatic coagulation easily occurs in the early stage of trauma, and the coagulation index (PLT, PTA, Fib) affects the demand for blood transfusion [16, 27]. In the process of building the CRT model, the variables corresponding to the root nodes are the most important, followed by the leaf nodes, which split in turn [20]. In addition to Hct and Fib, CRP is an important variable for predicting blood transfusion. Because CRP is an indicator of body stress, CRP stress increases after trauma, which can reflect the trauma severity [28]. In the process of establishing the XGBoost model, the more times the nodes are traversed, the more important the variables corresponding to the nodes are. The importance of variables is mathematically relevant, and whether they have clinical guiding value needs to be comprehensively analyzed in combination with clinical experience.

With the progress of science and technology, artificial intelligence methods have been widely used in the field of medicine [29–32]. There is considerable research on machine learning methods in trauma [33–35]. There has been considerable research on the prediction of massive blood transfusion, and the prediction accuracy of the decision tree algorithm is (0.695–0.814), [36, 37]. Machine learning (mostly neural networks) has been used in a large number of studies to predict the prognosis of trauma. Most studies have proven the benefits of machine learning methods, and the sensitivity-specificity difference ranges from 0.035 to 0.927 [38]. The neural network algorithm accuracy (98.7%) and specificity (51.5%) were the highest in predicting the survival rate of trauma patients [39].

Our research compares the traditional statistical methods with the machine learning decision tree algorithm, and the decision tree algorithm has outstanding advantages: (1) Most of the data in the real world are incomplete (missing key indicators) and noisy (numerical errors/anomalies). Artificial intelligence can allow cases with missing data or outliers to be retained by interpolation and other methods. The larger the number of cases, the more meaningful the statistical results; (2) The XGBoost algorithm is widely used in medicine, and the prediction performance is good [40, 41], 3) The model can reconstruct more effective features from the training process of blood transfusion big data, which can be used to predict the blood transfusion volume of patients to make the model have stronger generalization ability and reduce overfitting; (4) Using the difference between the prediction results and the training data for training, with the gradual increase in the data quantity, the accuracy improves in the iterative process, which ensures the incremental learning characteristics of the model; and (5) Currently, doctors are widely used to make blood transfusion decisions by combining various physiological parameters, symptoms and clinical experience. Our research uses a large quantity of historical data as a reference on the basis of doctors’ rich clinical experience, establishes a mathematical model, and adjusts the output of multiple experiments to obtain the best results. It has more practical value for primary hospitals or inexperienced doctors. In the future, with the increase in the data quantity, the model can be optimized by self-learning, and the prediction performance will continuously improve. The artificial intelligence mathematical model we constructed can be transformed into intelligent prediction software, which can be connected with ambulances and doctors’ working computers and can be widely used in clinics as an auxiliary tool to provide blood transfusion decision support for clinicians. The mature prediction model we constructed has wide applicability, and the data from other medical institutions can be retrained and applied to clinical practice. In the future, we can work with multiple medical centers to verify the predictive performance and universal applicability of the model.

Limitations of the study: The study data are available from the authors upon reasonable request and with permission from the Chinese National Engineering Laboratory for Medical Big Data Application Technology. Therefore, the database is not completely open, and cannot be disclosed. The artificial intelligence method is used to construct the mathematical model, which is limited to the fact that the data quantity is not large enough, and the accuracy of the model needs to be improved, but with the increase in the data quantity and the continuous optimization of the model, the prediction accuracy of the model will gradually improve. The variables extracted from unstructured text information are limited, which does not improve the performance of the model, so how to use the effective information to improve the prediction efficiency of the model is the direction of our future research. Some of the patients in our trauma database were transferred to our hospital from primary hospitals after emergency treatment (including blood transfusion), so the number of patients requiring emergency massive blood transfusion was relatively small, but it does not affect the establishment and application of the model. Our model can make decisions on whether a transfusion is based on changing, real-time vital signs and laboratory data in the process of trauma development. With large blood loss after trauma, complications such as hypothermia, acidosis and coagulation dysfunction easily occur, and the amount of plasma and platelet transfusion has an effect on the RBC demand. However, our model includes indicators that reflect these symptoms, so the effects of these complications and blood components on erythrocyte demand have been considered.

## Conclusions

The traditional LR has the best classification ability when using non-invasive parameter prediction in the intelligent evaluation of post-traumatic blood transfusion demand, but it is only suitable for data analysis and cannot be used in clinical applications. The classification performance of the intelligent prediction model constructed by the decision tree algorithm is not inferior to that of the traditional LR method. With the increase in data quantity, the accuracy of the model improves in the iteration process, and the prediction performance continuously improves, which is conducive to clinical application and wide promotion.

## Supplementary Information

**Additional file 1: Table 1.** Examples for 10 patients with their features. **Table 2.** Binary logistic regression analysis for predicting transfusion with non-invasive detection parameters.

## Data Availability

All authors had full access to all the data in the study.

## Abbreviations

AB: actual bicarbonate
AUC: area under the curve
APTT: activated partial thromboplastin time
CI: confidence interval
CRP: C-reactive protein
CRT: classification and regression tree
DBP: diastolic blood pressure
Fib: fibrinogen
Hb: haemoglobin
Hct: haematocrit
HR: heart rate
IL-6: interleukin-6
INR: international standardized ratio
ISS: injury severity score
K: potassium
Lac: lactate
LR: logistic regression
PCO_2_: partial pressure of carbon dioxide
pH: potential of hydrogen
PLT: platelet count
PO_2_: partial pressure of oxygen
PT: prothrombin time
PTA: prothrombin activity
R: respiration
RBC: red blood cell
SB: standard bicarbonate
SBP: systolic blood pressure
SD: standard deviation
SI: shock index
SpO_2_: blood oxygen saturation
T: temperature
TCO_2_: total carbon dioxide
Trauma_loc: trauma location
VD: vasoactive drugs
XGBoost: eXtreme gradient boosting
24 h RBC: the volume of 24-h red blood cell transfusion
