# Supplementary material for: Intelligent prediction of RBC demand in trauma patients using decision tree methods
Source: Mil Med Res. 2021 May 24;8:33. doi: 10.1186/s40779-021-00326-3 (PMC8142481; doi:10.1186/s40779-021-00326-3)
Supplement: Supplementary file 1 — Additional file 1: Table 1. Examples for 10 patients with their features. Table 2. Binary logistic regression analysis for predicting transfusion with non-invasive detection parameters. [file 40779_2021_326_MOESM1_ESM.docx]

**Additional Table 1** Examples for 10 patients with their features

| No. | 1 | 2 | 3 | 4 | 5 | 6 | 7 | 8 | 9 | 10 |
| --- | --- | --- | --- | --- | --- | --- | --- | --- | --- | --- |
| Sex | Female | Male | Male | Female | Female | Male | Male | Male | Female | Female |
| Age (year) | 47 | 46 | 29 | 18 | 33 | 60 | 60 | 53 | 88 | 72 |
| Height (cm) | NA | NA | NA | NA | NA | 160 | NA | NA | NA | 145 |
| Weight (kg) | NA | NA | NA | NA | NA | 80 | NA | NA | NA | 77 |
| Non-invasive parameters |  |  |  |  |  |  |  |  |  |  |
| HR(beat/min) | 122 | 77 | 133 | 79 | 76 | 81 | 92 | 105 | 102 | 80 |
| R (beat/min) | 14 | 22 | 28 | 14 | 20 | 20 | 18 | 22 | 21 | 19 |
| SBP (mmHg) | 142 | 115 | 83 | 135 | 112 | 141 | 102 | 124 | 133 | 143 |
| DBP (mmHg) | 97 | 82 | 54 | 88 | 61 | 71 | 72 | 88 | 69 | 78 |
| SpO_2_ (%) | 99 | 99 | 98 | 100 | 99 | 94 | 89 | 97 | 86 | 98 |
| SI | 0.86 | 0.67 | 1.60 | 0.59 | 0.68 | 0.57 | 0.90 | 0.85 | 0.77 | 0.56 |
| T (℃) | NA | NA | 37.0 | 38.1 | 36.8 | 37.6 | 37.3 | 36.5 | 36.2 | 36.1 |
| Invasive detection parameters |  |  |  |  |  |  |  |  |  |  |
| Hb (g/L) | 107 | 67 | 88 | 67 | 79 | 88 | 62 | 160 | 122 | 153 |
| Hct (L/L) | 0.322 | 0.291 | 0.250 | 0.192 | 0.233 | 0.268 | 0.186 | 0.470 | 0.360 | 0.450 |
| PLT (×10^9^/L) | 165 | 287 | 246 | 46 | 166 | 160 | 57 | 251 | 230 | 161 |
| CRP (mg/L) | 1.59 | 0.76 | 6.70 | 31.50 | 0.20 | 3.60 | 11.50 | NA | 0.10 | 0.57 |
| IL-6 (pg/ml) | NA | NA | NA | 225.10 | 9.06 | 40.93 | 3.62 | NA | 68.40 | NA |
| PT (s) | 21.1 | 16.1 | 13.7 | 15.8 | 15.0 | 14.9 | 17.4 | 12.7 | 15.0 | 16.0 |
| APTT (s) | 54.4 | 35.7 | 45.9 | 33.6 | 31.0 | 32.5 | 47.0 | 31.6 | 33.6 | 36.2 |
| INR | 1.83 | 1.31 | 1.05 | 1.26 | 1.18 | 1.17 | 1.42 | 0.95 | 1.17 | 1.28 |
| PTA (%) | 44 | 67 | 93 | 70 | 77 | 78 | 59 | 109 | 78 | 69 |
| Fib (g/L) | 0.58 | 2.32 | 6.21 | 4.54 | 1.96 | 2.49 | 3.36 | 3.73 | 2.10 | 1.88 |
| pH | 7.32 | 7.32 | 7.46 | 7.41 | 7.31 | 7.43 | 7.45 | 7.42 | 7.44 | 7.43 |
| PO_2_ (mmHg) | 135 | 131 | 93 | 186 | 170 | 70 | 54 | 93 | 49 | 97 |
| PCO_2_ (mmHg) | 47 | 30 | 34 | 34 | 46 | 36 | 33 | 35 | 29 | 43 |
| TCO_2_ (mmol/L) | 25.6 | 16.4 | 25.2 | 22.6 | 24.6 | 25.0 | 23.9 | 23.8 | 20.6 | 29.8 |
| Lac (mmol/L) | 6.4 | 5.4 | 0.9 | 5.1 | 1.4 | 0.7 | 2.5 | 4.4 | 3.0 | 1.5 |
| AB (mmol/L) | 24.2 | 15.5 | 24.2 | 21.6 | 23.2 | 23.9 | 22.9 | 22.7 | 19.7 | 28.5 |
| SB (mmol/L) | 23.4 | 17.6 | 25.6 | 22.8 | 22.5 | 24.7 | 24.4 | 24.0 | 22.0 | 27.7 |
| K (mmol/L) | 3.9 | 4.0 | 3.6 | 3.7 | 4.1 | 3.6 | 4.5 | 3.3 | 3.6 | 3.9 |
| Endotracheal intubation | Y | N | N | Y | N | N | N | N | N | N |
| Vasoactive drugs | Y | N | N | Y | N | N | N | N | N | N |
| Diagnosis | Multiple injuries | Multiple injuries | Shoulder contusion | Multiple injuries | Stab wound | Multiple injuries | Multiple injuries | Car accident injury | Multiple injuries | Multiple injuries |
| Trauma location |  |  |  |  |  |  |  |  |  |  |
| Head and neck | Y | Y | N | N | N | Y | Y | Y | Y | N |
| Upper extremity | N | N | Y | N | N | N | N | N | N | N |
| Lower extremity | N | N | N | N | N | N | N | N | N | N |
| Chest and abdomen | Y | Y | N | Y | Y | Y | Y | N | Y | Y |
| Spine | N | N | N | N | N | N | N | N | Y | N |
| Pelvis | N | N | N | N | N | N | N | N | N | N |
| Trunk | N | N | N | N | N | N | N | N | N | N |
| Trauma severity classification | First level | First level | First level | First level | First level | First level | First level | First level | First  level | Second level |
| Trauma type |  |  |  |  |  |  |  |  |  |  |
| Open trauma | N | Y | N | N | Y | N | N | N | N | N |
| Blunt injury | Y | N | Y | Y | N | Y | Y | N | N | N |
| RBC volume (U) | 6 | 4 | 2 | 2 | 4 | 4 | 2 | 0 | 0 | 0 |
| 24h RBC (U) | 6 | 4 | 0 | 2 | 4 | 4 | 2 | 0 | 0 | 0 |
| Emergency department time (h) | 8.29 | 3.15 | 6.07 | 21.16 | 19.10 | 20.47 | 14.00 | 6.14 | 6.25 | 20.25 |

HR. Heart rate; R. Respiration; SBP. Systolic blood pressure; DBP. Diastolic blood pressure; SpO_2_. Blood oxygen saturation; T. Temperature; SI. Shock index; Hb. Haemoglobin; Hct. Haematocrit; PLT. Platelet count; CRP. C-reactive protein; IL-6. Interleukin-6; PT. Prothrombin time; APTT. Activated partial thromboplastin time; INR. International standardized ratio; PTA. Prothrombin activity; Fib. Fibrinogen; pH. Potential of hydrogen; PO_2_. Partial pressure of oxygen; PCO_2_. Partial pressure of carbon dioxide; TCO_2_. Total carbon dioxide; SPO_2_. Oxygen saturation; Lac. Lactate; AB. Actual bicarbonate; SB. Standard bicarbonate; K. Potassium; RBC. Volume of red blood cell transfusion; 24h RBC. The volume of 24-hour red blood cell transfusion; Y. Yes; N. No; NA. Not available

**Additional Table 2** Binary logistic regression analysis for predicting transfusion with non-invasive detection parameters

| Variable | *OR* | 95%CI | *P*-value |
| --- | --- | --- | --- |
| T | 0.843 | 0.626 - 1.137 | 0.262 |
| Trauma location | 18.371 | 4.019 - 83.931 | 0.000 |
| SI | 3.463 | 1.763 - 6.801 | 0.000 |

T. Temperature; SI. Shock index; *OR*. Odds ratio
